# Refractory Takayasu’s Arteritis with Severe Coronary Involvement—Case Report and Literature Review

**DOI:** 10.3390/jcm12134394

**Published:** 2023-06-29

**Authors:** Claudia Oana Cobilinschi, Elena Grădinaru, Ioana Săulescu, Nicolae Cârstea, Simona Caraiola, Andra Rodica Bălănescu, Daniela Opriș-Belinski

**Affiliations:** 1 Department of Rheumatology and Internal Medicine, Carol Davila University of Medicine and Pharmacy, 050474 Bucharest, Romania; ioana_oprisan@yahoo.com (I.S.); scaraiola@yahoo.com (S.C.); balanescu.andra@gmail.com (A.R.B.); danaopris0103@yahoo.com (D.O.-B.); 2 Department of Rheumatology and Internal Medicine, Sf Maria Clinical Hospital Bucharest, 011172 Bucharest, Romania; elena.gradinaru@rez.umfcd.ro; 3 Department of Interventional Cardiology, Ares Excellency Centers, 021967 Bucharest, Romania; nicucarstea@yahoo.com; 4 Department of Internal Medicine, Colentina Clinical Hospital, 020125 Bucharest, Romania

**Keywords:** Takayasu’s arteritis, angina pectoris, coronary involvement, myocardial infarction, angioplasty, in-stent restenosis, revascularization techniques

## Abstract

This report presents the case of a female patient diagnosed with Takayasu arteritis from childhood, with severe, refractory coronary involvement, leading to two acute coronary syndromes and multiple anginous episodes. Consequently, the patient suffered aorto-bicarotid bypass two times, multiple interventional procedures with stent implantation, balloon angioplasty, and up to ten repeated in-stent restenosis that required reinterventions, despite being on maximal immunosuppressive treatment. In recent years, various studies have been reported that aim to best characterize this particular type of vascular damage and to indicate optimal therapeutic options for treatment. The latter should be based on the activity of the underlying disease; however, no reliable markers are available in TA. The management of TA patients with coronary involvement continues to be a challenge and requires both drug and interventional techniques to avoid life-threatening events.

## 1. Introduction

Takayasu’s arteritis (TA) is a rare large vessel vasculitis, mainly affecting the aorta and its branches. It is a chronic granulomatous-type inflammation that increases patients’ morbimortality through occlusive or aneurysmal lesions.

The diagnosis of TA can sometimes be challenging in the absence of specific biomarkers, but it is based on a thorough patient medical history and clinical examination, inflammatory markers like erythrocyte sedimentation rate (ESR) and C-reactive protein (CRP), and imaging techniques to detect vessel inflammation [1].

The updated 2022 American College of Rheumatology ACR/EULAR classification criteria for TA includes as mandatory the age of the patient to be under 60 and the evidence of vasculitis on imaging while adding points for clinical or imaging criteria. The clinical domain counts one point for the female gender and a difference of over 20 mmHg in systolic blood pressure between arms as well as two points for each of the following: angina or ischemic heart pain, limb claudication, vascular bruit at auscultation, reduced or no pulse in upper limbs, pulse abnormalities or tenderness of the carotid arteries. The imaging criteria account for points depending on the number of involved arterial sites (one, two, three, or more), symmetric arterial involvement, abdominal aorta, and renal or mesenteric injury. A score of a minimum of five points is required for TA classification [2].

The use of imaging for aiding TA diagnosis has been recently proposed in European recommendations, and that is because in TA-suspected patients, the magnetic resonance imaging (MRI) is the optimal tool to assess mural inflammation or changes in the vessel lumen. This technique is preferred since it has no irradiation risks for the young TA population, and it can be reliable to detect wall active inflammation. An alternative to MRI is the use of positron emission tomography (PET), computed tomography (CT), or ultrasound. However, the latter is of limited use for the investigation of the thoracic aortic segment. Conventional angiography is no longer a preferred method for diagnosis because of the risks and invasiveness of the procedure. Imaging can also be used to assess TA activity in case of disease flares, but it is not routinely recommended if remission is stable [3].

TA can be identified in both adults and children with different disease patterns and vascular distribution. TA typically affects young patients, under the age of 40, with a predominance of female gender that have a dominant involvement of the thoracic aorta as opposed to males that display inflammation of the abdominal aorta and branches. TA is significantly more frequent in Asia and Japan has the highest reported prevalence of up to 40 million. In Europe, the prevalence is estimated between 4.7–33 per million [4].

The pathogenesis is yet not fully understood but the latest publications indicate that a mixture of cytotoxic lymphocytes and mast cells are dominant in the granulomatous inflammatory infiltrate that is responsible for vessel lesions in TA by first damaging the media and then forming a reparatory fibrotic tissue [5]. The pathogenic process firsts targets the vasa vasorum of the adventitia with the formation of the inflammatory infiltrate due to the activation of innate (dendritic cells, natural T killer cells) and adaptative immunity (T and B lymphocytes). Along with the cell gathering, pro-inflammatory mediators (interleukins IL-6,8,9,18,19, tumor necrosis factor TNF) are increased in TA patients, both in serum and vascular tissues and it appears their levels are higher during active phases of the disease. Environmental factors, such as molecular mimicry for certain microorganisms, also play a key role in TA but the mechanism is yet not fully understood [6].

The inflammatory process in TA causes the intimal layer of the vessel to become hyperplastic and the adventitia to thicken, leading to ischemia due to tissue perfusion impairment. The distribution of the inflammation is usually segmental but rarely it can damage the entire vessel wall, leading to stenosis, occlusion, or consequent dilation. These changes are distributed on the subclavian arteries, brachiocephalic trunk, and carotid or vertebral arteries, but territories of the abdominal aorta can also be affected [7]. Coronary artery involvement is rarely noted in TA, with a frequency estimated at 9–11%, with the coronary ostia being the dominantly affected site. In patients with life-threatening vasculitis, the treatment aims at reducing inflammation through corticosteroids, immunosuppressants, and interventional or surgical treatment. Despite the latter, up to one-fifth of TA patients exhibit a refractory form of the disease with critical vascular lesions [8]. 

The localization and degree of arterial inflammation in TA vary widely. Until recently, the Numano classification based on angiographic findings was able to identify six TA subgroups (aortic arch, ascending, thoracic or descending aorta, abdominal aorta or renal vessels, and combined subtype) [9]. More recently, another classification divided patients into clusters, as follows: cluster 1 includes the involvement of the abdominal aorta and branches, cluster 2 aortic arch and branches, and cluster 3 asymmetric and lesser arterial injury [6]. 

In several cases, the diagnosis of TA is set when vascular damage is already present, thus treatment results can be suboptimal.

Treatment in TA includes two dimensions: agents that can rapidly reduce systemic inflammation to present vessel damage and remodeling and interventional procedures that can resolve consequences of disease like thrombus formation, tissue hypoperfusion, or ischemia.

Current standard-of-care in TA is the initiation of glucocorticoids 40–60 mg daily with progressive tapering within one year and the addition of a conventional synthetic antirheumatic drug like methotrexate, azathioprine, leflunomide, or mycophenolate mofetil, and in case they fail, cyclophosphamide can be an option. If the disease is still active, refractory, or has flares, glucocorticoid dose can be adjusted, and biological therapy (tocilizumab or anti-TNF drugs) can be attempted. Interventional procedures are indicated in case vascular inflammation leads to persistent symptoms or it can cause complications, but the time and type of intervention depend on the arterial site, disease activity at the time of intervention, involved organs, and other potential risk factors [10]. 

We present a case of a female patient diagnosed with TA from childhood, with refractory coronary involvement, suffering aorto-bicarotid bypass two times, and multiple interventional procedures with stent implantation, balloon angioplasty, and up to ten repeated intrastent restenosis that required reinterventions, while being on maximal immunosuppressive treatment. We aim to disseminate essential information on the importance of coronary site involvement in large vessel vasculitis and raise awareness among clinicians of the need to investigate in case of a patient’s evocative symptoms, so that prompt therapeutic decision can be made. 

Taking into consideration the scarcity of data regarding coronary site involvement in TA, we performed an overview of the literature to better define this finding. Knowledge about refractory TA cases will further increase the chance for optimized management in future patients. 

## 2. Case Presentation

**Figure 1:**
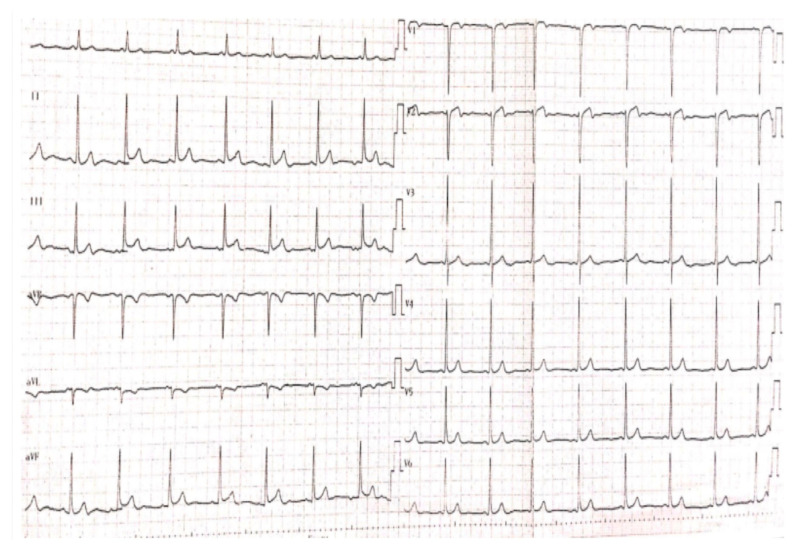
EKG showing ST segment elevation in the inferior territory.

**Figure 2:**
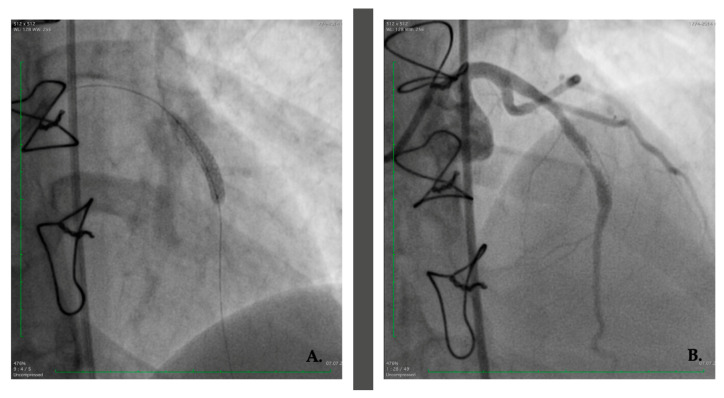
First patient restenosis treated with a drug-eluting stent in a bare-metal stent (**A**) Drug-eluting stent implantation after bare-metal stent restenosis. (**B**) Right anterior oblique cranial angulation view: in-stent restenosis on the descending anterior artery; patent left main and circumflex artery.

We present the case of a 26-year-old female patient who is under rheumatology follow-up since 2015 when she was referred to our department after recent cardiology management of an anterior myocardial infarction with ST segment elevation (STEMI), as suspected on an initial electrocardiogram (Figure 1). A 90% stenosis of the anterior descending coronary artery (LAD) was identified for which an angioplasty was performed per primam with bare-metal stent (BMS) placement. Four months later, the patient developed angina for which a 70% intrastent restenosis was incriminated, so that a drug-eluting stent (DES) was implanted. A year later, the latter was reoccluded, so that a balloon angioplasty was made (Figure 2). Dual antiplatelet therapy (DAPT) was recommended after testing for aspirin and clopidogrel response.

The arteriographic examination of the aortic arch showed occlusion of the brachiocephalic trunk from its origin, and occlusion of the subclavian arteries but permeable renal arteries. Repeated venous Doppler ultrasound showed no abnormal findings.

A thorough anamnesis revealed that the patient was diagnosed with type I Takayasu arteritis (TA) at the age of 13 when she was admitted for fatigue, weight loss, and persistent headache. At the time, she had no pulse in her axillary, brachial, and radial arteries, a blood pressure difference of over 30 mmHg between upper limbs, and vascular bruits confirmed during auscultation of her carotid, subclavian, and renal arteries. She displayed no inflammatory syndrome. After ruling out infectious, congenital, or miscellaneous diseases, the diagnosis of Takayasu was established, according to pediatric TA classification criteria. Patient had no evocative family history or prior medical events. Due to subclavian arterial occlusion, significant stenosis of the left common carotid artery, brachiocephalic trunk, and vertebral vessels, and an aortic bicarotid bypass with Gore-Tex was performed at her juvenile age. Corticosteroids and cyclophosphamide (six cycles of intravenous treatment followed by oral administration) were initiated given the severity of vessel involvement, but later disrupted because of apparent disease remission. 

At the age of 20, she delivered a healthy baby through C-section, with no further complications during pregnancy and no chronic immunosuppressive treatment at the time. 

After only six months from her 2015 evaluation, the patient was readmitted to the cardiology department for effort angina and a non-ST elevation myocardial infarction (NSTEMI) was confirmed, leading to pharmacologically active intrastent balloon angioplasty. 

Two years later, the patient displayed chest pain upon small effort and the exercise stress echocardiography was positive for ischemia in the LAD territory, with hypokinesia of the interventricular septum. After three months, another intrastent restenosis was identified on coronarography and balloon angioplasty was again performed. Moreover, the examination added the presence of an atheromatous plaque on the circumflex artery. 

During the following year, the patient had negative tests for ischemia, including electrocardiographic (EKG) exercise stress tests, and no dyskinesia or arrhythmia on cardiac ultrasound. 

**Figure 3:**
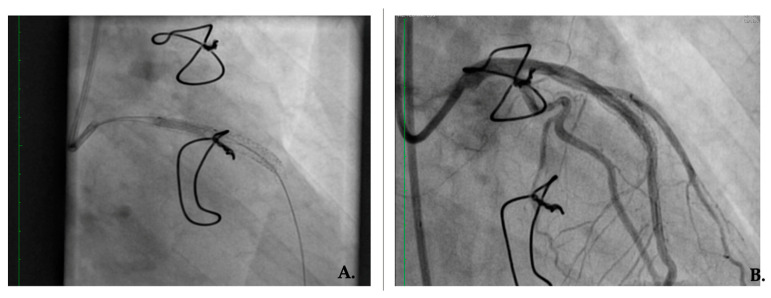
Predilatation and treatment with a drug-eluting balloon. (**A**) Scoreflex balloon predilatation for in-stent restenosis; (**B**) Good final result after scoreflex balloon and drug-eluting balloon dilation for in-stent restenosis.

In 2019, the patient underwent another pharmacologically active balloon angioplasty for focal 90% restenosis on LAD. However, stress tests (clinical, EKG, echocardiogram) were positive eight months later in the LAD territory, with apex hypokinesia and intense precordial pain. Two months later, another intervention was necessary for the same intrastent re-occlusion and the placement of two pharmacologically active stents on the LAD I and LAD II segments (Figure 3).

**Figure 4:**
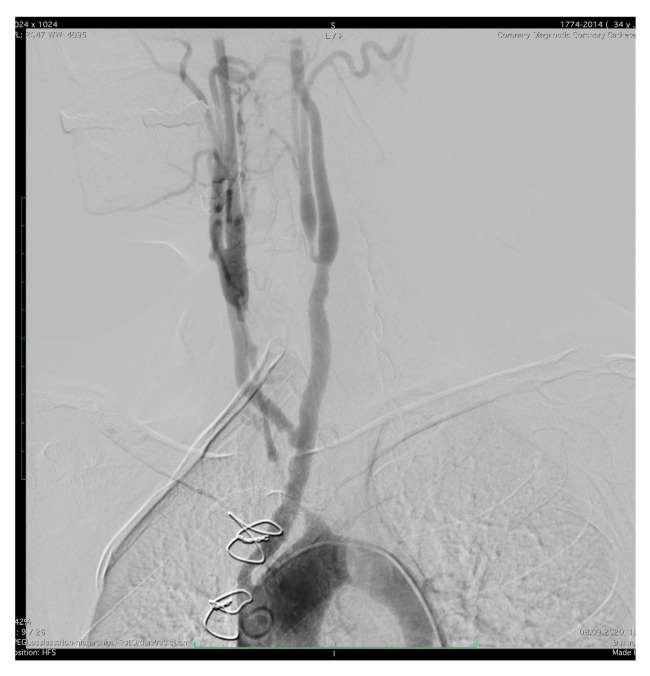
Aortography of the ascending aorta and aortic arch. Anteroposterior projection: occlusion of the right and left subclavian artery, occlusion of the left common artery, patent-bi-carotid bypass with 50% stenosis of the right branch proximal.

A year later, in 2020, the patient suffered from another NSTEMI through 80% restenosis of the LAD II stent, and further on, multiple episodes of unstable angina due to serial 70% restenosis of the LAD I, for which non-compliant and pharmacologically active balloon angioplasty was managed. Five months later, the patient underwent surgery for aortocoronary bypass, respectively, left anterior descending artery (LAD) and aorta with inversed independent saphenous venous grafts (Figure 4). The patient was discharged with favorable postoperative evolution and up to present disease has been controlled, with no additional ischemic events.

Cardiological treatment was stable since 2015 and included beta-blockers, angiotensin-enzyme converting inhibitors, diuretics, DAPT, statin, and later add-on ezetimibe. Under this chronic treatment, patient had controlled blood pressure values upon regular check-ups in the rheumatology ward (systolic blood pressure under 130 mmHg measured in both lower limbs). 

**Figure 5:**
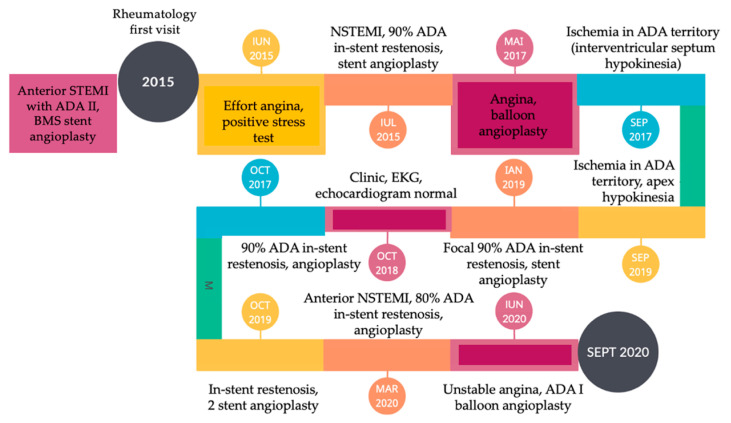
Chronological synopsis of patient’s cardiac events and consequent interventions.

The patient’s cardiological sequence of interventions is briefly summarized in a graphic view in Figure 5.

Starting at the age of 26, in 2015, the patient was under continuous rheumatological follow-up and received corticosteroids (prednisone 1 mg/kg body weight equivalent) and immunosuppressants, namely cyclophosphamide, adding four more intravenous cycles. However, ischemic events while on treatment, led to switching the patient to azathioprine with a gradual dose increase until 2018. Persistent vascular manifestations imposed the initiation of methotrexate for two more years, which was later changed with mycophenolate mofetil up to 3 g daily in 2020 when surgery was undergone.

During these years, the patient was on variable corticosteroid doses, depending on her cardiac symptoms and follow-up. She was started on intravenous cyclophosphamide, adding up to ten cycles, followed by oral azathioprine 150 mg daily. Non-response led to the initiation of methotrexate 15 mg weekly, rapidly switching to mycophenolate 2.5 g under which remission was attained. Considering the severity of the disease with vital organ involvement, biological therapy was discussed as an off-label option, but the patient was unwilling to initiate treatment.

Upon regular rheumatological monitoring, biological investigations, namely inflammatory markers like erythrocyte sedimentation rate (ESR) and C-reactive protein (CRP), were repeated, with no increase of the reference value levels during active phases of the disease. The renal and hepatic functions were normal, and so was the blood count. Optimal laboratory values were maintained following the CABG procedure.

Up to the present, the patient had no novel cardiac symptoms, and follow-up revealed a stable vascular state with no need for further interventional procedures. She is currently under a stable dose of corticosteroids and mycophenolate mofetil.

## 3. Materials and Methods

**Figure 6:**
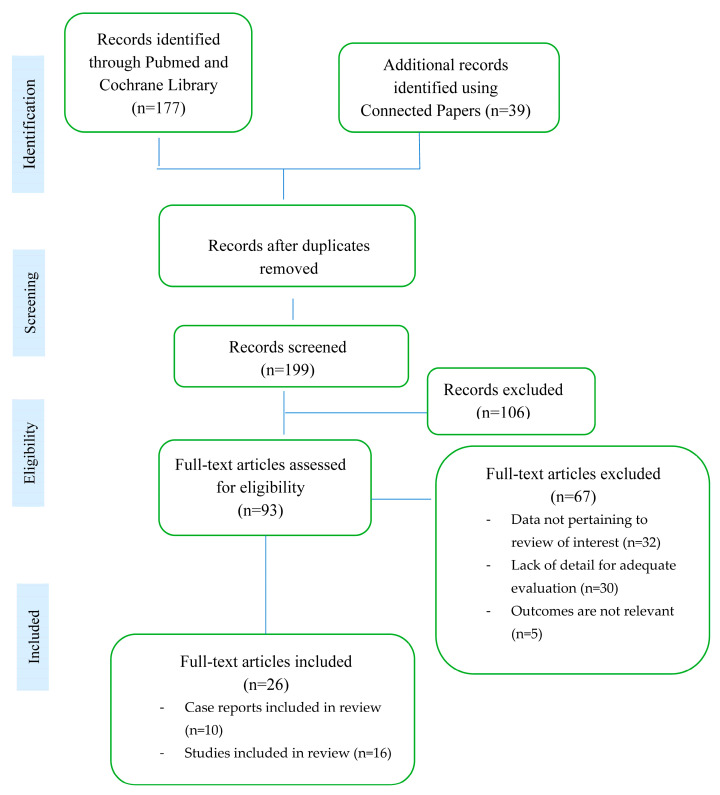
Results of systematic search and selection process.

A literature search was done to capture the relevant data about several areas of interest associated with Takayasu arteritis (TA). Medical subject headings (MeSH) terms like “Takayasu arteritis”, “coronary arteries”, “angina pectoris”, “myocardial infarction”, “angioplasty”, and “in-stent restenosis” were used. The search was performed in PubMed and Cochrane Library, where two independent observers identified relevant sources for this review from 2000–2023 (Figure 6).

Titles and abstracts were screened first. The articles that depicted cases related to Takayasu in child or adolescent populations, did not match at least two keywords, or used languages other than English were excluded. The lists of these articles were checked for additional records.

After an abstract selection, the corresponding full-text article was evaluated, and selected to be used if eligible. The eligibility was determined based on references to Takayasu arteritis with a specific focus, namely coronary artery damage associated with TA, the frequency of disease onset with symptoms such as angina or myocardial infarction, and the different techniques of interventional or surgical revascularization. 

## 4. Results

The selected literature was represented by case reports (38.46%) and studies (61.53%), suggesting increased interest in understanding the involvement of the coronary arteries within TA as well as in choosing an appropriate treatment to improve clinical outcomes of this specific category of patients.

Firstly, it has been observed that coronary artery involvement is quite common in TA [2,7]. Secondly, most of the papers show that the election site of involvement in TA is the coronary ostium. Depending on the type of lesion, three types of involvement have been noted in the autopsies of TA patients: occlusion or stenosis of the coronary ostia and the proximal segments (type 1), focal or diffuse coronary arteritis, in the form of “skip” lesions or can spread diffusely to all epicardial branches (type 2), and coronary aneurysms (type 3) [11]. The most encountered lesion is type 1. 

Regarding the cardiac symptoms of TA with coronary artery involvement, stable/unstable angina pectoris [12,13,14], dyspnea on exertion [14,15], and retrosternal chest pain that indicates the presence of acute myocardial infarction [13,16,17,18] have been identified in the literature.

Regarding inflammatory markers used in TA to assess disease activity and dictate treatment options, erythrocyte sedimentation rate (ESR) and high-sensitivity C-reactive protein (hsCRP) are most commonly used. Furthermore, one study showed that hsCRP can predict adverse cardiovascular events in patients with TA with coronary artery involvement [19]. One retrospective analysis showed that biological inflammation at the time of revascularization increases the risk of vascular complications that warrant reinterventions [20].

The treatment of patients with coronary artery stenosis associated with TA is individualized depending on the disease activity and the severity of cardiac manifestations that can be vital. Thereby, optimal management requires immunosuppressive treatment and various revascularization techniques. If active inflammation is identified, preferably on imaging, patients should receive immunosuppressive therapy before revascularization except in cases of emergency surgery. Corticosteroids are the first-line treatment to control active inflammation, with suggested initial dosages of 0.5–1 mg/kg/day. If necessary, corticosteroids are used in combination with immunosuppressants, usually methotrexate [21]. Concerning revascularization techniques for coronary stenosis secondary to TA, the most used are PCI, PCI with drug-eluting stents, and CABG [22]. PCI is to be elected as a revascularization strategy over CABG in poor surgical candidates or patients who prefer a nonsurgical treatment. Drug-eluting stents are considered more effective compared with bare-metal stents. For the latter, a consistent number of studies report a lower incidence of reocclusion of the coronary artery [15]. However, despite the procedure, coronary angioplasty and drug-eluting stent insertion are followed by a high rate of restenosis [23]. One study indicates that CABG is an option to choose in patients who require emergency revascularization. Otherwise, PCI may be an alternative to CABG after patients reach remission of the disease with medical therapy [24]. After the revascularization intervention, restenosis may occur even in the absence of clinical symptoms, thereby follow-up is crucial [25,26].

## 5. Discussion

Takayasu arteritis (TA) is described as a large vessel vasculitis, targeting the aorta and its branches. The case depicts a young female patient diagnosed with TA from childhood, complicated with a challenging coronary artery involvement within her vasculitis that required several interventions and adjustment of immunosuppressive therapy. Although coronary artery damage seemed to be a rather rare site involved in TA [15], some articles described the opposite [11,22]. 

The coronary arteries are estimated to be affected in up to a third of TA patients by developing stenosis, but even a higher percentage can display valvular dilatation or regurgitation. TA with coronary involvement leads to a poorer prognosis because of the non-specificity of symptoms and delay in diagnosis. Patients can display dyspnea upon effort, angina, heart palpitation, myocardial infarction, or even death. In adults, coronary involvement appears to be linked to age at disease onset and its duration, but also with the atherogenic profile of patients, such as triglycerides, cholesterol fractions, and homocysteine. Traditional cardiovascular risks of TA patients also contribute to atherosclerosis [27]. It appears that the mechanism is multicausal since coronary vessels suffer from the process of chronic inflammation and vasculitis but also from early atherosclerosis. Vessel stenosis occurs when the inflammation of the intimal layer progresses and causes hyperplasia, and there is a contraction of the aorta media and adventitia tunics. Later on, thrombosis formation either from inflammation or atherosclerosis is favored, increasing the risk for myocardial infarction. The percentage of coronary lesions in pediatric TA is significantly higher than in the adult population, ranging from 39–55%, and the vessel injury can be indolent with no clinical symptoms or electrocardiographic changes, only identifiable by ultrasound [28]. 

Similar to the presented case that involved the proximal part of the left coronary artery, literature data related to the results of angiography also suggest that the most frequent sites involved are represented by the coronary ostium and the proximal segment of the coronary arteries [29]. Although in some articles, left main coronary artery (LMCA) appears to be the most commonly affected location [15,22], a recent systematic review describes the right coronary as the most commonly affected [25]. Type 1 lesion, namely the coronary ostia and proximal coronary segments, is the most frequent. In addition to the chronic inflammatory process, an important factor in the etiology of lesions is also played by premature atherosclerosis as in the case described above [11]. 

Regarding gender distribution in coronary artery involvement among TA, although women are predominantly affected, males tend to have more severe coronary stenosis and a higher risk for long-term mortality than females [30].

Cardiac symptoms are always present; however, a retrospective study shows that angina pectoris is the most common initial symptom and for this reason, coronary artery involvement should be suspected especially in young women with TA [31]. Acute myocardial infarction is a rare form of presentation [17].

Assessing the disease activity is challenging, since no tool has been standardized in TA. Disease activity is an important prognostic factor in patients with TA and coronary stenosis because it is associated with progression [32]. It involves the evaluation of signs and symptoms, biological inflammatory syndrome and imaging monitoring (digital subtraction angiography (DSA), computed tomography angiography (CTA), magnetic resonance imaging (MRI), ultrasonography, and positron emission tomography with radio-labeled glucose (FDG-PET). 

Imaging plays an important role in monitoring disease evolution, especially in cases where biochemical inflammation is absent. Available recommendations state that non-invasive imaging is preferred to catheter angiography for disease activity monitoring and therapeutic decisions and should be done regularly, in addition to clinical evaluation. CT/MRI angiography or FDG-PET are routinely recommended for identifying vessel extension. Still, no clear indication is given for coronary artery evaluation [33]. Recent data suggests the use of PET/MRI to better describe vessel wall inflammation and disease remission, progression, relapses in a non-irradiating way [34]. Moreover, it appears that future somatostatin receptor (SST2) PET/MRI will offer more accurate details on arterial inflammation and atherosclerotic lesions, since it can detect macrophages, pericytes, and adipocytes involved in the inflammation [35]. 

In the absence of high inflammatory markers, the presented patient still developed in-stent restenosis and de novo myocardial infarction, thus, therapeutic decisions were guided upon clinical manifestations or positive cardiological tests confirming ischemic events. Nevertheless, assessment remains a challenge because of the scarcity of clinical symptoms in some patients and the fact that evidence suggests that inflammatory markers (ESR, hsCRP) cannot reliably distinguish active from inactive disease. For this reason, the use of these markers during asymptomatic periods can lead to the false assumption that the patient is in remission while there is ongoing active fibrosis and progressive stenosis [36]. Their lack of specificity led to the need to identify novel biomarkers like interleukin (IL)-6, -8, -18, and tumor necrosis factor (TNF)-alpha. Higher levels of IL-6 and IL-18 have been found in active TA patients, displaying a higher risk for restenosis. Moreover, determining interleukin levels might be useful when initiating biological therapy such as tocilizumab, an anti-IL-6 agent [37].

The alternative to using inflammatory markers for assessing disease activity in TA is angiography, but the main concern is related to being an invasive and irradiating procedure [36].

Regarding the treatment of coronary artery stenosis associated with TA, the optimal method has yet to be determined. The 2018 EULAR update on large vessel vasculitis emphasizes the use of corticosteroids which can be associated with drug-modifying antirheumatic drugs (DMARDs) like methotrexate, azathioprine, leflunomide, or cyclophosphamide in non-responders [10,21,27]. Biological treatments like tocilizumab or anti-TNF agents are recommended in refractory forms of TA, with results coming from two randomized controlled trials. However, their use in regard to cardiological interventions, surgery, or specific points to consider in vital coronary involvement has not yet been published. In 2021, the American College of Rheumatology (ACR) published evidence-based recommendations for large vessel vasculitis, including TA. ACR expert opinion states the need for high-dose glucocorticoids upon diagnosis, favoring the oral route in adult TA patients. If disease activity is maintained despite glucocorticoids, immunosuppressive agents can be added and, if required, anti-TNFs. The latter have a stronger recommendation than anti-IL-6 agents due to higher clinical experience. 

Regarding monitoring disease activity and vascular involvement, the ACR recommends evaluating inflammatory markers and regular monitoring. However, if novel progression of vascular lesion occurs or if inflammatory markers increase with no evidence of clinical activity, monitoring is preferred to augmentation of immunosuppression [33]. 

One of the ACR recommendations states that if signs of organ suffering are present, escalation of immunosuppressants is the first step to take. However, specific mention goes to coronary artery involvement that urges prompt surgical intervention to prevent tissue necrosis. No indication of a specific revascularization technique for coronary involvement is mentioned in the ACR guidelines [33]. 

The most used revascularization techniques for coronary stenosis secondary to TA are PCI, PCI with drug-eluting stents, and CABG [22,32]. PCI may be a more beneficial revascularization strategy than CABG for poor surgical candidates or patients who prefer a nonsurgical treatment. Drug-eluting stents are being considered more effective compared with bare-metal stents, with many studies reporting a lower incidence of restenosis in the coronary artery [15]. However, coronary angioplasty and drug-eluting stent insertion are affected by a high rate of restenosis. A study has shown that arterial stiffness measured through brachial–ankle pulse wave velocity can identify TA patients with DES who have a high risk for in-stent restenosis and Major Adverse Cardiovascular Events (MACE) [23]. CABG in patients with TA brings many difficulties because the internal mammary artery used successfully in patients with coronary stenosis of atherosclerotic etiology can develop vasculitis and graft stenosis in TA patients, thus, vein grafts (saphenous vein grafts) are more preferred to arterial grafts [25]. A multicenter study that followed up patients with coronary artery involved in TA who underwent either PCI or CABG showed that PCI group had a significantly higher incidence of restenosis (63.2%) vs. (25%) during the median follow-up of 101 months. It also showed that PCI had a very high rate of in-stent restenosis in patients without corticosteroids and CABG may be a preferred treatment option [31].

Disease activity has a great influence in terms of revascularization techniques. Coronary intervention should be avoided during the active phase of the disease unless the presence of an acute life-threatening condition outweighs the risk of intervention. Re-revascularization rate was lower in patients who underwent revascularization after the disease was stabilized [32]. 

Regarding the choice of the revascularization procedure in patients with active disease, one study indicates that CABG is an option to choose in the case of patients who require emergency revascularization. Otherwise, PCI may be an alternative to CABG after the patients reach the remission state with drug therapy [24]. Another option in the case of patients with an uncontrolled inflammatory TA disease awaiting revascularization is represented by drug-eluting stents; these stents can function as a bridge therapy until the disease is controlled, possibly improving patency rates of future definite vascular interventions [11]. More insight is needed on when to perform revascularization techniques and a comparison between procedures. 

Using PCI for the aortic lesions led to optimal results and rupture avoidance due to cover stent use, and so were the iliac arteries. For mesenteric and renal arteries, covered stents are also beneficial. However, coronary lesions were not successful with PCI and bypass surgery needs to be considered if restenosis occurs. 

Very few recent publications aimed at assessing outcomes of percutaneous intervention in 942 patients with TA, summing 2450 arterial lesions, most of them stenotic or occlusive (2365) followed by aneurysms or dissections (85). Results are more than encouraging for PCI, showing it is safe and durable in most TA lesions. Early success was reached in more than 90% of lesions, late success in 84.5% within 50 months of follow-up, and only 17% of lesions suffered complications that were resolved. Post-PCI occlusion appears more frequent than after surgical bypass, but repeated PCI was able to resolve the lesion in the cohort of the abovementioned study. Revascularization through repetitive PCI is considered safer since it is a minor intervention usually through the same site as opposed to the bypass graft that needs to be reassessed for qualitative perfusion. It is not uncommon for the first PCI to lead to a restenosis; thus, patients should be informed that multiple similar processes might be required. This study brings forward a rather individualized choice when using balloon angioplasty of stenting, as follows: balloon angioplasty should be used per primam in young patients with recent disease when lesions are expected to be soft, with no significant vascular remodeling and with a non-ostial location. An elective stent can be preferred in older patients with a chronic injury where arteries have significant mural thickening or are located at the ostial area or the aorta. The present study indicated that CBAG is less safe in TA because of potential dissection, rupture, or pseudoaneurysm formation [38]. 

Main results of selected review publications are summarized in Table 1.

**Figure 7:**
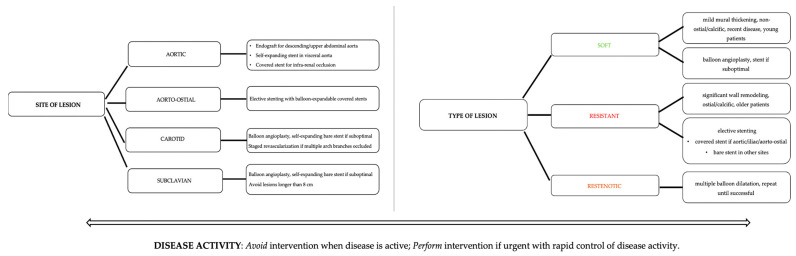
Approach to PCI in TA, adapted from Joseph et al.

Joseph et al. propose an approach to PCI in TA, depending on the type and site of lesion and disease activity, summarized below in Figure 7 [38]:

The incidence of major adverse cardiovascular events in TA patients who underwent revascularization at the active stage was higher in the PCI group than in the CABG group during a median of 41.0 months follow-up. For patients in an active stage, the risk of MACE was significantly lower in patients than those without therapy with prednisone [24]. 

There is limited data about dietary intake and interventions as adjuvant therapies in connective tissue disorders, mainly on n-3 polyunsaturated and short-chain fatty acids, taking into consideration comorbidities that characterize these conditions [39]. Other macronutrients, such as proteins in the pediatric population, showed no implication in vasculitis recurrence [40,41], although they are important modulators of growth and inflammation [42,43].

A summary of all the studies and case reports discussed previously according to the publication domain is shown in Table 1.

In the presented patient, the aorto-carotid bypass was the only remaining option and was only possible by using venous grafts, given that the mammary arteries were unavailable due to subclavian arteries occlusion. In a non-TA patient, the patency of venous bypass is estimated at five years [44], so she will need close monitoring to check for functionality. In four out of ten interventions, the patient remained asymptomatic, with no angina or dyspnea prior to hospitalization. The follow-up is crucial after revascularization procedures because many cases of coronary artery restenosis in Takayasu’s arteritis occur without detectable cardiac symptoms [25,26].

The presented case is an exhibit that TA can involve life-threatening vascular territories that need prompt therapeutic action, including aggressive immunosuppression and interventional revascularization techniques to limit disease damage accrual. 

Multidisciplinarity plays an essential role in TA detection, investigation, and treatment since patients can have a display of multiorgan involvement depending on the affected arterial territories. Rheumatologists, pediatricians, radiologists, cardiologists, and interventional physicians should collaborate to follow standard-of-care in TA patients to improve long-term outcomes. Since TA affects young patients, including pediatric populations, regular and lengthy follow-up is mandatory. The latter is significantly more useful since in some cases there is a quiescent disease progression in the absence of symptoms of biochemical inflammation that clinicians should identify and halter before vascular damage arises. 

## 6. Conclusions

Takayasu arteritis can become a life-threatening vasculitis when affecting coronary arteries, leading to a poor patient outcome. The case depicts a unique TA patient who suffered from ten successful in-stent restenosis and double bypass, given her severe, refractory disease. Screening for risk factors for coronary involvement together with methods of prevention and control can improve patients’ outcomes. A proactive type of monitoring is essential even in pseudo-asymptomatic patients, given that inflammatory markers do not accurately correlate to disease activity. The therapeutic approach in TA includes optimal immunosuppression as well as interventional and surgical techniques in order to limit further vascular damage. Critical vascular involvement in TA requires prompt diagnosis and multidisciplinary management.

## Figures and Tables

**Table 1:** Summary of main results in selected review publications.

Publication Domain	Author, Year	Article Type (Case Report /Study)	Summary
Clinical	Grayson P. C. et al., 2012, 2022 [2,7] Acha M. R et al., 2007 [11]	Study Study	Coronary artery involvement is common in TA Types of involvement: occlusion or stenosis of the coronary ostia and the proximal segments (type 1), focal or diffuse coronary arteritis (type 2) and coronary aneurysms (type 3)
	Sun T. et al., 2013 [29]	Study	The most frequent sites involved are the coronary ostium and the proximal segment of the coronary arteries
	Yang Y. et al., 2017 [31]	Study	Males tend to have more severe coronary stenosis and a higher risk for long-term mortality
	Cobilinschi C. et al., 2021 [27]	Case report	Coronary involvement is linked the atherogenic profile of patients
	Xu Y. et al., 2023 [28]	Study	The percentage of coronary lesions is pediatric TA is significantly higher than in the adult population
	Park J.S. et al., 2009 [12] Yokota K. et al., 2012 [13] Soni M. R. et al., 2012 [14] Lee H.K. et al., 2011 [15] Zhou S. et al., 2021 [16] Zhang T. et al., 2019 [17] Mihailovici A.R et al., 2018 [18]	Case report Case report Case report Case report Case report Case report Case report	Common symptoms in TA with coronary involvement: angina pectoris, dyspnea on exertion, retrosternal chest pain as acute myocardial infarction
Biological	Wang X. et al., 2016 [19]	Study	HsCRP can predict adverse cardiovascular events in TA with coronary artery involvement
	Saadoun D. et al., 2012 [20] Wang H. et al., 2020 [32]	Study Study	Biological inflammation at revascularization increases the risk of vascular complications with reinterventions Disease activity predicts progression in patients with TA and coronary stenosis
	O’Connor T.E. et al., 2014 [33]	Study	Inflammatory markers are not reliable to distinguish active from inactive disease
Drug therapy	Huang Z. et al., 2021 [21]	Study	Corticosteroids are used in combination with immunosuppressants, preferably before revascularization
Surgical therapy	Kuijer A. et al., 2015 [22] Wang X et al., 2015 [23] Wang X et al., 2017 [24]	Case report Study Study	Revascularization techniques for coronary stenosis are PCI, PCI with drug-eluting stents and CABG Coronary angioplasty and drug-eluting stent insertion have a high risk of restenosis PCI is an alternative to CABG after patients reach remission of the disease with medical therapy
	Yuan S. M., 2020 [25] Al-Hulaimi N. et al., 2001 [26]	Study Case report	Restenosis may occur even in the absence of clinical symptoms
	Ci W. et al., 2022 [30] Pathadan A.P et al., 2021 [34]	Study Study	Determining interleukin levels can be of use when initiating biological therapy

## Data Availability

Not applicable.
